# The Cause of Death

**Published:** 1882-10

**Authors:** 


### ARTICLE VI.

### *The Cause of Death.*

We know, almost for certain, how death takes place in all fatal diseases; *i, e.*, we say, that an individual dies, either by the brain, heart or lungs. In reality, however, we are still ignorant how death is brought about, how the machinery comes to a sudden stand-still, what extinguishes with the last flickering of the final expiration, the vital spark forever. The brain may be extirpated, and animal life has been observed to be still going on: the heart may apparently cease beating, and yet the existence of the being is not finally ended; respiration is to all appearances not filling and emptying the air vessels any more, but still more than vegetation keeps up the complicated machinery, he spark of life still lingers.

What, then, is it that finally causes death? Does it not look very much like the sudden action of a powerful poison, which so thoroughly and at once disintegrates the great circulating, life-giving and life-preserving fluid, the blood, that the nerve centres and their existence, and force the organism to cease every motion, like an engine deprived of its steam?

Experiments that have lately been made, and which we have to thank for the discovery of the *Ptomaineo*, may tend to lead us a step further in the solving of the question before us. A. Gautiei has made these ptomaines a special study. It has been demonstrated that many organic substances, the fibrin in the blood, the urine, and certain compounds of tissues, when decomposing, give rise to alkaloids—called ptomaines—which are powerful poisons. This discovery we owe mainly to Selmi. But Gautier has done more. He thought that if such alkaloids are formed on the decomposition of organic substances, why may not, in the course of the common change of tissues, if the latter is deviating from its true course, also such ptomaines be called into existence.

The result of these investigations fully confirmed his theory. A disciple of G. Pouchet succeeded in obtaining from the normal urine a fixed and easily crystallizable alkaloid, the hydrochloric compounds of which crystallize, as well as those with gold chlorid, and platinum chlorid. And this alkaloid has been found to be exceedingly poisonous; in most minute doses it causes stupor, tetanus and death, within the shortest time imaginable, stopping the heart is systole. This very quality relegates this alkaloid of the urine to the ptomaines, for the toxic effect of which the stoppage of the heart in systole is most characteristic. A mixture of ferrum-chlorid, and ferro-cyanide of potassium colors this alkaloid blue, as it does all ptomaines. Gautier was able to isolate from the poison of two poisonous snakes (Trigorrocephalus and Naja) two similar alkaloids.

Gautier proved also the intensely poisonous action of the human saliva on birds. He evaporated twenty grms. in the water bath, dried the 0.25 grm., weighing residue, and dissolved it in warm water. This solution injected into a small bird, under the skin, caused stupor, dilatation of the pupil, increased frequency of respiration, and in all cases, death. He was able to isolate an alkaloid also out of the human saliva. This alkaloid gave the same color reaction as the other ptomaines.

Another characteristic quality of these ptomaines is, that they form salts with most of the vegetable alkaloids, as morphia, codeia, atropia, veratria aconitia, hyoscyamia, eserin, and others. The ptomaines are very easily oxidizable, and may be formed during the normal change of tissues, even without the presence of oxygen.

What may all these investigations, and their astonishing results, not explain. If a hypodermic injection of some vegetable alkaloid accidentally is made into a larger vein, and a fatal result the consequence, under all the symptoms which we used to ascribe to the entrance of air, may not the real cause of death be found, perhaps, in the sudden formation of these poisonous animal alkaloids ? May not many other similar occurrences, which to-day are clouded in mystery as yet, find a similar interpretation ? May not, at the time of the dissolution of the body, a similar process turn on, forever the flame of life ? Is it urea, in uræmia, that causes the fatal uræmic symptoms, or does the presence of urea in certain quantities, and under given conditions, produce the formation of these highly poisonous animal alkaloids ?

The subject is one of great interest to the medical profession. We mentioned in one of our late numbers, when speaking of the pure keratin pre pared from the eggshell membrane, and its easy transformation into leucin and tprosin, the " *multum e parvo* " of nature, and here we have a still more glaring proof, with what few means nature —as with the magic touch of the mystic wand—achieves the greatest results.—*Editorial in Med. and Surg. Reporter*.

